# Can AI modeling of protein structures distinguish between sensor and helper NLR immune receptors?

**DOI:** 10.1111/nph.70391

**Published:** 2025-07-15

**Authors:** AmirAli Toghani, Raoul Frijters, Tolga O. Bozkurt, Ryohei Terauchi, Sophien Kamoun, Yu Sugihara

**Affiliations:** ^1^ The Sainsbury Laboratory University of East Anglia Norwich Research Park Norwich NR4 7UH UK; ^2^ Department of Biotechnology Rijk Zwaan Breeding B.V. Fijnaart 4793 the Netherlands; ^3^ Department of Life Sciences Imperial College London London SW7 2AZ UK; ^4^ Iwate Biotechnology Research Center Kitakami Iwate 024‐0003 Japan; ^5^ Crop Evolution Laboratory Kyoto University Muko Kyoto 617‐0001 Japan

**Keywords:** AlphaFold, functional specialization, NLR immune receptor, plant–pathogen interactions, protein structure

Nucleotide binding and leucine‐rich repeat (NLR) proteins are intracellular immune receptors that occur across all kingdoms of life but are particularly highly diversified in plants (Barragan & Weigel, 2021). In plants, NLRs that carry a coiled‐coil (CC) domain at their N termini are the most phylogenetically widespread class. Following pathogen recognition, CC‐NLR proteins oligomerize into pentameric or hexameric pore‐like complexes (Wang *et al*., 2019; Förderer *et al*., 2022; Zhao *et al*., 2022; Liu *et al*., 2024; Madhuprakash *et al*., 2024). These complexes, known as resistosomes, are a defining feature of NLRs that execute the immune response; some of them are known to translocate to cellular membranes and trigger immune responses such as calcium influx and hypersensitive cell death (Duggan *et al*., 2021; Contreras *et al*., 2022; Ibrahim *et al*., 2024). The prevailing model is that the funnel‐shaped structure of CC‐NLR resistosomes inserts into membranes and is required for executing the cell death and immune response (Wang *et al*., 2019; Adachi *et al*., 2019b; Förderer & Kourelis, 2023). This funnel‐shaped structure is formed by the N‐terminal α1 helix, which is a structurally dynamic region that is difficult to resolve using cryo‐electron microscopy (cryo‐EM) (Förderer *et al*., 2022; Zhao *et al*., 2022; Liu *et al*., 2024; Madhuprakash *et al*., 2024).

NLRs function as singletons, pairs, or networks (Adachi *et al*., 2019b; Contreras *et al*., 2023a). Singleton NLRs can detect pathogens and execute hypersensitive cell death and immune responses, while paired and networked NLRs have subfunctionalized into sensor (pathogen detection) and helper (immune execution, also known as ‘executors’) NLRs that carry distinct biochemical activities. Paired NLRs often originate from distinct phylogenetic clades yet function together, making them more difficult to classify based on phylogenetic relationships compared with NLR networks (Kourelis *et al*., 2021; Contreras *et al*., 2023a). Sensor and helper NLR pairs are often genetically clustered, and some sensors have noncanonical integrated domains (IDs) that function in pathogen sensing and are absent in helper NLRs (Białas *et al*., 2018; Marchal *et al*., 2022). The presence of IDs provides a useful *in silico* criterion for distinguishing sensor NLRs from helpers. In addition, *c*. 20% of plant CC‐NLRs have a conserved sequence motif, called MADA, in the N‐terminal α1 helix, and this motif has degenerated in some sensor NLRs of solanaceous plants (Adachi *et al*., 2019a). However, the structural basis underlying the functional specialization of CC‐NLRs into sensors and helpers remains unclear.

Since its release in 2024, AlphaFold 3 (AF3) has significantly advanced structural modeling of NLR immune receptors (Abramson *et al*., 2024; Ibrahim *et al*., 2024; Madhuprakash *et al*., 2024). Notably, AF3 is capable of modeling protein structures with oleic acids serving as a proxy for cellular membranes (Abramson *et al*., 2024). This capability allows researchers to predict structures of regions that have been notoriously difficult to resolve experimentally, such as the funnel‐shaped structure of resistosomes (Ibrahim *et al*., 2024; Madhuprakash *et al*., 2024).

Here, we use AF3 to generate hypotheses about the functional roles of genetically linked NLRs. We leveraged AF3 to explore the structural diversity of sensor and helper oligomers of a curated set of CC‐NLRs consisting of experimentally validated NLR pairs in rice (Pikm, Pii, and Pia), their orthologs (PIK5/6‐NP, Pi5‐3/1, and Pias), and two previously cloned NLR pairs in barley (RPG5/HvRGA1 and RGH2/3) (Supporting Information Table S1; Fig. S1). As in previous studies (Ibrahim *et al*., 2024; Madhuprakash *et al*., 2024), we used the oligomerizing domains of the NLR proteins, from the N terminus to the end of the NB‐ARC domain, and performed AF3 predictions of 5× and 6× stoichiometries with 50 oleic acids and using three different seed values (1, 2, and 3). We then compared the sensor and helper predicted template modeling (pTM) and ipTM scores (Figs 1a,b, S2–S4; Table S2). In both pentameric and hexameric configurations, helper NLRs consistently exhibited higher pTM scores than sensor NLRs.

**Fig. 1:**
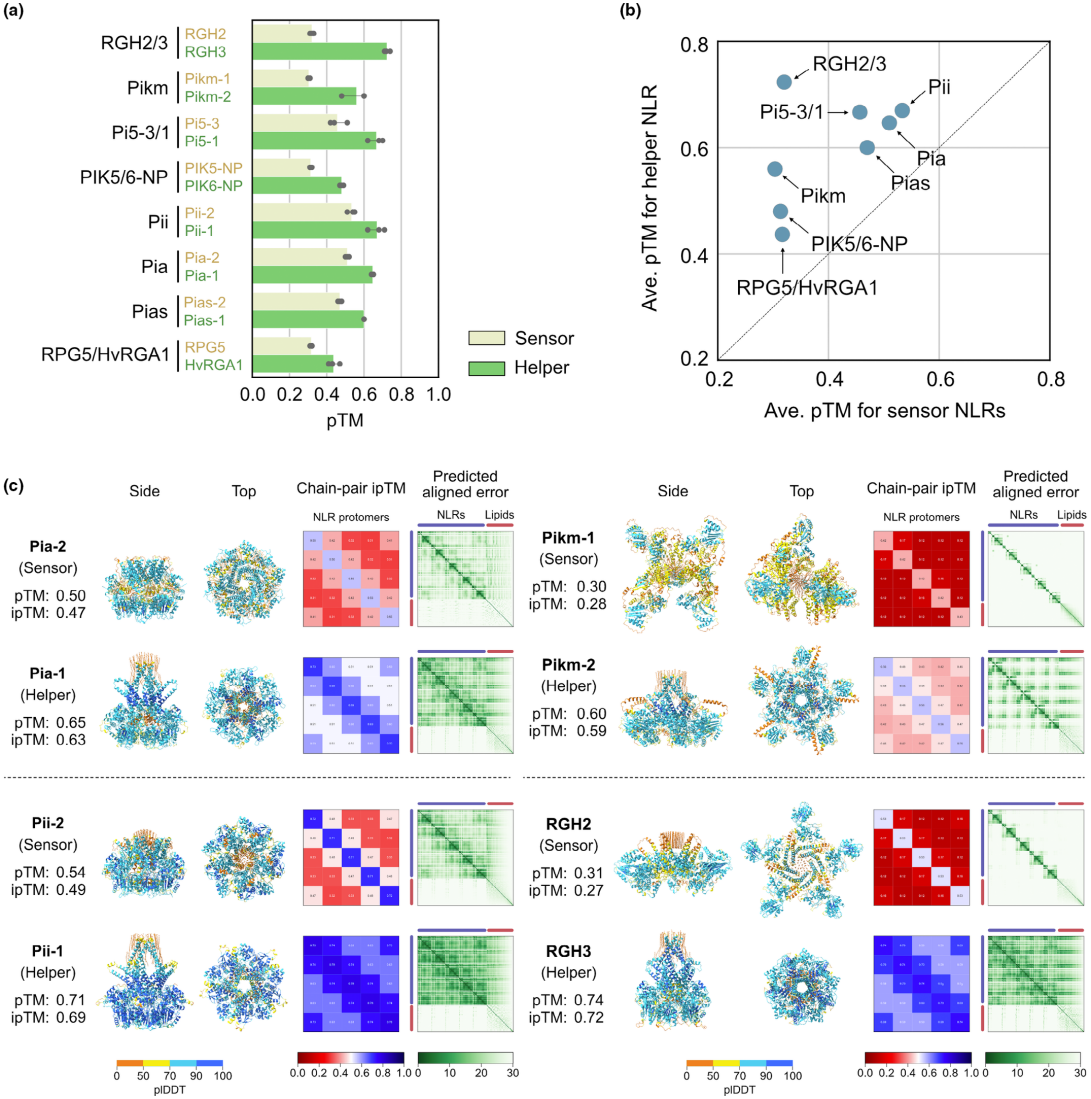
Helper nucleotide binding and leucine‐rich repeats (NLRs) produce higher AlphaFold 3 (AF3) confidence scores than their paired sensors. (a) Bar plot comparing sensor and helper predicted template modeling (pTM) scores in pentameric AF3 predictions. The amino acid sequences of the oligomerizing domains of the NLR proteins, from the N terminus to the end of the NB‐ARC domain, were used for the prediction. The pentameric structures were modeled with 50 oleic acids using three different seed values. (b) Scatter plot comparing sensor and helper pTM scores in pentameric AF3 predictions. The resulting pTM scores were averaged across three seed values for each sensor and helper NLR. (c) Pentameric AF3 structures of the four representative NLR pairs. The structures predicted using seed value 1 were visualized with oleic acids (orange) using ChimeraX (Meng *et al*., 2023).

In addition to the AF3 confidence scores, we also examined the AF3 structures to identify distinct structural patterns between sensor and helper NLRs (Figs 1c, S5, S6). Helper NLRs consistently formed funnel‐shaped structures that exposed the N‐terminal α1 helices in contrast to sensor NLRs. This observation aligns with previous studies demonstrating that sensor NLRs cannot execute the immune response on their own and may have lost the capacity to oligomerize into resistosome‐like structures (Adachi *et al*., 2019b; Contreras *et al*., 2022). Sensor NLRs sometimes formed resistosome‐like structures but with low confidence scores (Figs 1c, S5, S6). These observations further indicate that AF3 can capture the structural characteristics of sensor and helper NLRs.

We then analyzed whether the presence of the MADA motif could classify sensors or helpers in the curated NLR pairs using the MADA HMM (hidden Markov model) in Adachi *et al*. (2019a) (Table S3). The helpers of Pikm and PIK5/6‐NP contained the MADA motif with HMM scores exceeding the cutoff value of 10, while the other sensors and helpers did not. Based on these results, only Pikm and PIK5/6‐NP could be classified according to the presence of the MADA motif, whereas the remaining NLR pairs could not be classified, indicating that the structure‐based classification is more robust than the sequence‐based classification and highlights the utility of AF3 for NLR classification.

We extended the analyses to rice putative paired NLRs. Based on Stein *et al*. (2018), we extracted 10 rice CC‐NLR pairs that: are genetically linked in head‐to‐head orientations; belong to distinct phylogenetic clades; and carry a full N‐terminal CC domain (Figs 2a, S1; Tables S4–S6). In five of the 10 pairs, one of the NLRs carries an ID annotation and is presumed to be the sensor. The putative helpers (without ID annotation) had higher AF3 pTM and ipTM scores than sensors (with ID annotation) for these pairs (Figs 2b, S7–S9; Table S7). These results further confirm the application of AF3 for functional classification as noted with the eight previously characterized pairs. Notably, Pair‐13 is identical to the rice pair PIK5/6‐NP, in which PIK5‐NP is known as a sensor with an ID that is not annotated by InterProScan (Białas *et al*., 2021; Kourelis *et al*., 2021). Nonetheless, AF3 successfully classified them into helper or sensor based on the confidence scores, which indicates that AF3 overcomes the limitations of sequence‐based annotation methods.

**Fig. 2:**
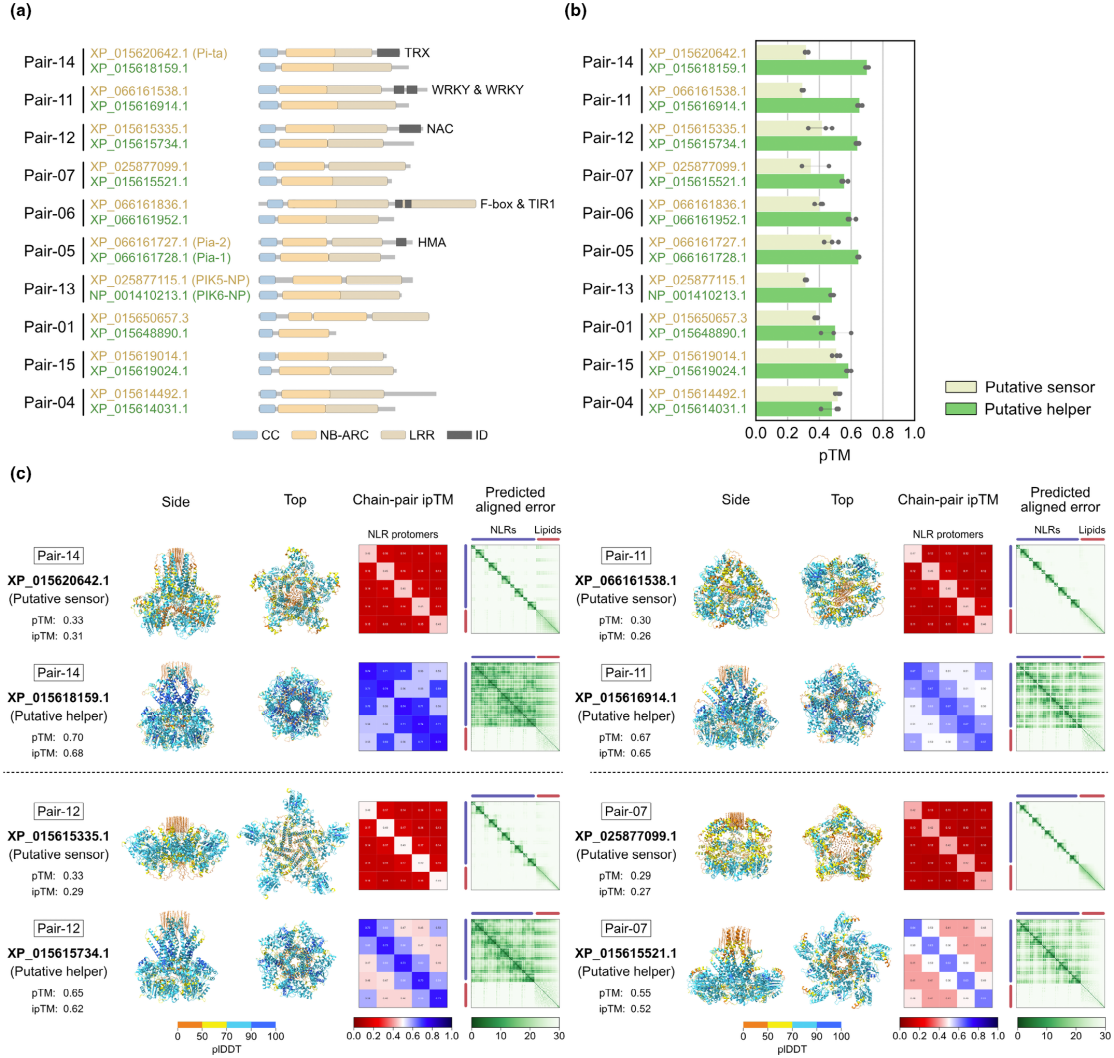
Nucleotide binding and leucine‐rich repeats (NLRs) without integrated domain (ID) exhibit higher AlphaFold 3 (AF3) confidence scores than NLRs with ID in rice putative paired NLRs. (a) Domain architectures of 10 rice NLR pairs described by Stein *et al*. (2018). The domains were annotated with NLRtracker (Kourelis *et al*., 2021) and visualized with refplantnlR (https://github.com/JKourelis/refplantnlR). (b) Bar plot comparing putative sensor and helper pTM scores in pentameric AF3 predictions. The amino acid sequences of the oligomerizing domains of the NLR proteins, from the N terminus to the end of the NB‐ARC domain, were used for the prediction. The pentameric structures were modeled with 50 oleic acids using three different seed values. Putative sensors and helpers were assigned based on the average predicted template modeling scores of pentameric and hexameric structures, with a putative sensor having the lower average score and a putative helper having the higher average score. (c) Pentameric AF3 structures of the four representative NLR pairs described by Stein *et al*. (2018). The structures predicted using seed value 1 were visualized with oleic acids (orange) using ChimeraX (Meng *et al*., 2023).

After the initial submission of this study, Guo *et al*. (2025) reported that two genetically linked NLR genes, Pm5e (sensor) and RXL (Rx‐CC‐like) (helper), arranged in a head‐to‐head orientation, function together as a genetically paired CC‐NLR module conferring powdery mildew resistance in wheat (Guo *et al*., 2025). Pm5e lacks an ID annotation, and both Pm5e and RXL have atypical domain architectures, characterized by an unusual CC domain in Pm5e and a truncated NB‐ARC domain in RXL (Guo *et al*., 2025). We tested whether AF3 can also distinguish between this experimentally validated wheat RXL/Pm5e pair, in addition to the rice and barley NLRs described previously. In accordance with the previous results, AF3 produced higher pTM and ipTM scores in both pentameric and hexameric predictions for the helper NLR RXL (Fig. S10; Tables S8, S9). These findings further highlight the utility of AF3 in predicting sensor and helper NLRs.

Why did sensor NLRs exhibit lower AF3 confidence scores than helper NLRs? Previous studies proposed a model suggesting that paired NLRs evolved from singleton NLRs (Adachi *et al*., 2019b). In this model, singleton NLRs, which can detect pathogens (sensor) and execute hypersensitive cell death (helper), subfunctionalized into sensor or helper NLRs (Adachi *et al*., 2019b; Contreras *et al*., 2023a). This subfunctionalization results in the loss of cell death activity in sensor NLRs and the loss of pathogen perception activity in helper NLRs. Since the formation of resistosomes with funnel‐shaped structures is essential for the induction of cell death and other immune responses, the presence of stable resistosome and funnel‐shaped structures in helper NLRs, but not in sensor NLRs, supports the previously proposed model. Taken together, the lower AF3 confidence scores observed in sensor NLRs may reflect their evolutionary divergence and functional specialization. Moreover, these results may indicate that sensor NLRs are intrinsically unable to oligomerize on their own, as observed in the sensor NLR Rx (Contreras *et al*., 2022).

Another notable observation is that the α1 helices of helper NLRs (Pia‐1, Pias‐1, and RGH3), which lack the MADA motif, formed the funnel‐shaped structures in AF3 predictions. This raises new questions, such as whether they can actually form funnel‐shaped structures in their activated oligomers, whether specific non‐MADA sequence patterns exist in funnel‐shaped α1 helices, and what determines their ability to form funnel‐shaped structures. Cryo‐EM and large‐scale AF3 predictions of NLR oligomers will help address these questions in the future.

It should be noted that the AlphaFold predictions presented here for CC‐NLRs focus exclusively on activated resistosome‐like oligomers and do not address the resting states, which can range from monomers and homodimers to more complex assemblies (Wang *et al*., 2019; Ma *et al*., 2024; Selvaraj *et al*., 2024). Furthermore, we have not explored the possibility in this work that sensor and helper CC‐NLRs form hetero‐oligomeric resistosome complexes, as observed in some mammalian inflammasomes (Hu *et al*., 2015; Zhang *et al*., 2015). Our primary aim in this study was to evaluate AlphaFold as a predictive tool to classify NLRs, which are highly diverse, often numbering in the hundreds per plant genome and collectively accounting for *c*. 1% of all plant genes (Toghani *et al*., 2025). Future experimental and computational studies will be essential to illuminate the structure of sensor/helper complexes, which remain poorly understood.

Recently, researchers have applied AlphaFold to the study of plant–pathogen interactions (Ibrahim *et al*., 2023, 2024; Sugihara *et al*., 2023; Tamborski *et al*., 2023; Contreras *et al*., 2023b; Cruz *et al*., 2024; Madhuprakash *et al*., 2024; Selvaraj *et al*., 2024; Seong *et al*., 2024; Pai *et al*., 2025). Notably, a recent study on an NLR network in the dicot species *Lactuca sativa* (lettuce) reported a contrasting structural pattern in sensors (without any IDs) and helpers, and experimentally validated the sensor–helper relationships: Sensor autoactive mutants do not induce cell death on their own but can activate wild‐type helper NLRs to trigger cell death (Pai *et al*., 2025). These approaches using AF3 can be useful for characterizing NLR functions in gene clusters and for prioritizing candidate sensor and helper NLR genes. Moreover, such structural insights may help elucidate how the distinct structures of sensor and helper NLRs contribute to their functional specialization.

## Materials and Methods

### NLR annotation

NLRs were annotated using NLRtracker v1.0.3 (Kourelis *et al*., 2021) and InterProScan v5.67‐99.0 (Jones *et al*., 2014). Note that InterProScan did not annotate an ID of PIK5‐NP as previously reported (Kourelis *et al*., 2021). Therefore, we manually replaced the domain architecture of PIK5‐NP from ‘CNL’ to ‘CONL’ as it contains the HMA domain between the NB‐ARC (nucleotide‐binding adaptor shared by APAF‐1, R proteins, and CED‐4) and LRR (leucine‐rich repeat) (Białas *et al*., 2021). The domain architectures were visualized using refplantnlR (https://github.com/JKourelis/refplantnlR). The MADA motifs were analyzed using the HMM in Adachi *et al*., 2019a and Hmmer v.3.4 (http://hmmer.org) with the option ‘‐‐max’. The NLRtracker and HMMER outputs are archived on Zenodo (https://doi.org/10.5281/zenodo.15552925).

### Curation of NLR sequences

The sequences of curated NLR pairs were derived from either RefPlantNLR (Kourelis *et al*., 2021) or NCBI (National Center for Biotechnology Information) (Table S1). Regarding the NLR pairs described by Stein *et al*. (2018), the protein sequences of *Oryza sativa* cv Nipponbare (Oryza_sativa_vg_japonica.protein.fasta) were downloaded from the URL (https://doi.org/10.7946/P2FC9Z). Based on data S6 in Stein *et al*. (2018), the NLR pairs that are: genetically linked in head‐to‐head orientations; belong to distinct phylogenetic clades were extracted (Table S4). Using DIAMOND BlastP v.2.1.9 (Buchfink *et al*., 2021), the corresponding NLR sequences were identified from the NCBI RefSeq annotation of rice cultivar Nipponbare genome (GCF_034140825.1). The best‐hit sequences were summarized in Table S5 and confirmed to be genetically linked in a head‐to‐head orientation. Based on NLRtracker outputs, the CC‐NLR pairs, which carry a full N‐terminal CC domain, were retained (Table S6). Pair‐05, Pair‐13, and the sensor of Pair‐14 correspond to Pia, PIK5/6‐NP, and Pi‐ta, respectively (Tables S4, S6).

### AF3 prediction

Based on NLRtracker outputs, the amino acid sequences from the N terminus to the end of the NB‐ARC domain were extracted. Using the AF3 web server (https://alphafoldserver.com), the extracted sequences were modelled in both pentameric and hexameric configurations with 50 oleic acids using three different seed values (1, 2, and 3). For the wheat helper RXL, the full‐length amino acid sequence was used as input due to its atypical domain architecture (Guo *et al*., 2025). The input sequences and resulting models are archived on Zenodo (https://doi.org/10.5281/zenodo.15552925). Welch's t‐test was performed using the ‘ttest_ind’ function from the SciPy Python library, with the option ‘equal_var = False’. Regarding the NLR pairs described by Stein *et al*. (2018), we averaged the pTM scores of pentameric and hexameric configurations and assigned them as putative helpers or sensors based on higher or lower pTM scores, respectively (Table S7).

### Phylogenetic analysis

We built the tree of monocot paired NLRs with RefPlantNLR (Kourelis *et al*., 2021) and a set of 4936 NLR proteins from the NLRtracker output of 13 RefSeq proteomes, including two dicot species (*Arabidopsis thaliana* and *Solanum lycopersicum*) and 11 monocot species (*Zea mays*, *Triticum aestivum*, *Setaria viridis*, *Phragmites australis*, *Phoenix dactylifera*, *Oryza sativa*, *Musa acuminata*, *Lolium perenne*, *Hordeum vulgare* subsp. *vulgare*, *Brachypodium distachyon*, and *Asparagus officinalis*) (Toghani & Kamoun, 2024). More details on the phylogenetics analysis are available on GitHub (https://github.com/amiralito/Paired_NLR_AF3).

## Competing interests

SK and TOB receive funding from industry on NLR biology and have cofounded a start‐up company (Resurrect Bio Ltd) related to NLR biology. SK has filed patents on NLR biology.

## Author contributions

AT, RF, TOB, RT, SK and YS planned and designed the research. AT and YS performed data analysis, collection and interpretation. YS, SK and AT wrote the manuscript.

## Supporting information


**Fig. S1** Phylogenetic tree of monocot paired nucleotide binding and leucine‐rich repeat proteins (NLRs) with RefPlantNLR and 4936 NLR proteins from 13 RefSeq proteomes, including two dicot species and 11 monocot species obtained from Toghani *et al*. (2024).
**Fig. S2** Correlations between pentameric and hexameric AlphaFold 3 confidence scores and between predicted template modeling (pTM) and interface predicted template modeling (ipTM) scores for previously reported NLR pairs.
**Fig. S3** Comparisons of sensor and helper AlphaFold 3 scores in pentameric and hexameric configurations for previously reported NLR pairs.
**Fig. S4** Scatter plots comparing average sensor and helper scores in AlphaFold 3 predictions.
**Fig. S5** Pentameric AlphaFold 3 predictions for previously reported NLR pairs.
**Fig. S6** Hexameric AlphaFold 3 predictions for previously reported NLR pairs.
**Fig. S7** Comparisons of putative sensor and helper AlphaFold 3 scores in pentameric and hexameric configurations for NLR pairs described by Stein *et al*. (2018).
**Fig. S8** Pentameric AlphaFold 3 predictions for NLR pairs described by Stein *et al*. (2018).
**Fig. S9** Hexameric AlphaFold 3 predictions for NLR pairs described by Stein *et al*. (2018).
**Fig. S10** Pentameric and hexameric AlphaFold 3 predictions of the wheat NLR pair Pm5e (sensor) and RXL (helper).

**Table S1** List of previously reported NLR pairs.
**Table S2** Summary of AlphaFold 3 predictions for previously reported NLR pairs.
**Table S3** HMM scores of MADA motifs in previously reported NLR pairs.
**Table S4** List of rice NLR pairs described by Stein *et al*. (2018).
**Table S5** BLASTP results using NLRs from Stein *et al*. (2018) as queries against those in the NCBI RefSeq annotation as subjects.
**Table S6** Summary of NLRtracker outputs for rice NLR pairs from the NCBI RefSeq annotation of Nipponbare (GCF_034140825.1), corresponding to those described by Stein *et al*. (2018).
**Table S7** Summary of AlphaFold 3 predictions for rice NLR pairs from the NCBI RefSeq annotation of Nipponbare (GCF_034140825.1), corresponding to those described by Stein *et al*. (2018).
**Table S8** Summary of the wheat NLR pair Pm5e (sensor) and RXL (helper) sequences.
**Table S9** Summary of AlphaFold 3 predictions for the wheat NLR pair Pm5e (sensor) and RXL (helper).
Please note: Wiley is not responsible for the content or functionality of any Supporting Information supplied by the authors. Any queries (other than missing material) should be directed to the *New Phytologist* Central Office.

## Data Availability

The scripts and dataset used for the phylogenetic analysis are available on GitHub (https://github.com/amiralito/Paired_NLR_AF3). The other datasets are archived on Zenodo (10.5281/zenodo.15552925).
