# Supplementary material for: Can AI modeling of protein structures distinguish between sensor and helper NLR immune receptors?
Source: New Phytol. 2025 Jul 15;248(1):17–23. doi: 10.1111/nph.70391 (PMC12409091; doi:10.1111/nph.70391)
Supplement: Supplementary file 1 — Fig. S1 Phylogenetic tree of monocot paired nucleotide binding and leucine‐rich repeat proteins (NLRs) with RefPlantNLR and 4936 NLR proteins from 13 RefSeq proteomes, including two dicot species and 11 monocot species obtained from Toghani et al. (2024). Fig. S2 Correlations between pentameric and hexameric AlphaFold 3 confidence scores and between predicted template modeling (pTM) and interface predicted template modeling (ipTM) scores for previously reported NLR pairs. Fig. S3 Comparisons of sensor and helper AlphaFold 3 scores in pentameric and hexameric configurations for previously reported NLR pairs. Fig. S4 Scatter plots comparing average sensor and helper scores in AlphaFold 3 predictions. Fig. S5 Pentameric AlphaFold 3 predictions for previously reported NLR pairs. Fig. S6 Hexameric AlphaFold 3 predictions for previously reported NLR pairs. Fig. S7 Comparisons of putative sensor and helper AlphaFold 3 scores in pentameric and hexameric configurations for NLR pairs described by Stein et al. (2018). Fig. S8 Pentameric AlphaFold 3 predictions for NLR pairs described by Stein et al. (2018). Fig. S9 Hexameric AlphaFold 3 predictions for NLR pairs described by Stein et al. (2018). Fig. S10 Pentameric and hexameric AlphaFold 3 predictions of the wheat NLR pair Pm5e (sensor) and RXL (helper). [file NPH-248-17-s002.pdf]

New Phytologist

***New Phytologist* Supporting Information**

Article title: **Can AI modelling of protein structures distinguish between sensor and helper NLR immune receptors?**

Authors: AmirAli Toghani[1], Raoul Frijters[2], Tolga O. Bozkurt[3], Ryohei Terauchi[4,5], Sophien Kamoun[1]*, Yu Sugihara[1,4]*

[1] The Sainsbury Laboratory, University of East Anglia, Norwich Research Park, NR4 7UH, United Kingdom

[2] Rijk Zwaan Breeding B.V., Department of Biotechnology, Fijnaart, 4793, The Netherlands

[3] Department of Life Sciences, Imperial College London, London, SW7 2AZ, United Kingdom

[4] Iwate Biotechnology Research Center, Kitakami, Iwate, 024-0003, Japan

[5] Crop Evolution Laboratory, Kyoto University, Muko, Kyoto, 617-0001, Japan

*Corresponding authors: sophien.kamoun@tsl.ac.uk (S.K.) and yu.sugihara@tsl.ac.uk (Y.S.)

Article acceptance date: 24 June 2025

The following Supporting Information is available for this article:

Fig. S1 Phylogenetic tree of monocot paired NLRs with RefPlantNLR and 4,936 NLR proteins from 13 RefSeq proteomes, including two dicot species and 11 monocot species obtained from Toghani et al., 2024.

Fig. S2 Correlations between pentameric and hexameric AlphaFold 3 confidence scores and between pTM and ipTM scores for previously reported NLR pairs.

Fig. S3 Comparisons of sensor and helper AlphaFold 3 scores in pentameric and hexameric configurations for previously reported NLR pairs.

Fig. S4 Scatter plots comparing average sensor and helper scores in AlphaFold 3 predictions.

Fig. S5 Pentameric AlphaFold 3 predictions for previously reported NLR pairs.

Fig. S6 Hexameric AlphaFold 3 predictions for previously reported NLR pairs.

Fig. S7 Comparisons of putative sensor and helper AlphaFold 3 scores in pentameric and hexameric configurations for NLR pairs described by Stein et al., 2018.

Fig. S8 Pentameric AlphaFold 3 predictions for NLR pairs described by Stein et al., 2018.

Fig. S9 Hexameric AlphaFold 3 predictions for NLR pairs described by Stein et al., 2018.

Fig. S10 Pentameric and hexameric AlphaFold 3 predictions of the wheat NLR pair Pm5e (sensor) and RXL (helper).

See separate Excel file:

Table S1 List of previously reported NLR pairs.

Table S2 Summary of AlphaFold 3 predictions for previously reported NLR pairs.

Table S3 HMM scores of MADA motifs in previously reported NLR pairs.

Table S4 List of rice NLR pairs described by Stein et al., 2018.

Table S5 BLASTP results using NLRs from Stein et al., 2018 as queries against those in the NCBI RefSeq annotation as subjects.

Table S6 Summary of NLRtracker outputs for rice NLR pairs from the NCBI RefSeq annotation of Nipponbare (GCF_034140825.1), corresponding to those described by Stein et al., 2018.

Table S7 Summary of AlphaFold 3 predictions for rice NLR pairs from the NCBI RefSeq annotation of Nipponbare (GCF_034140825.1), corresponding to those described by Stein et al., 2018.

Table S8 Summary of the wheat NLR pair Pm5e (sensor) and RXL (helper) sequences.

Table S9 Summary of AlphaFold 3 predictions for the wheat NLR pair Pm5e (sensor) and RXL (helper).

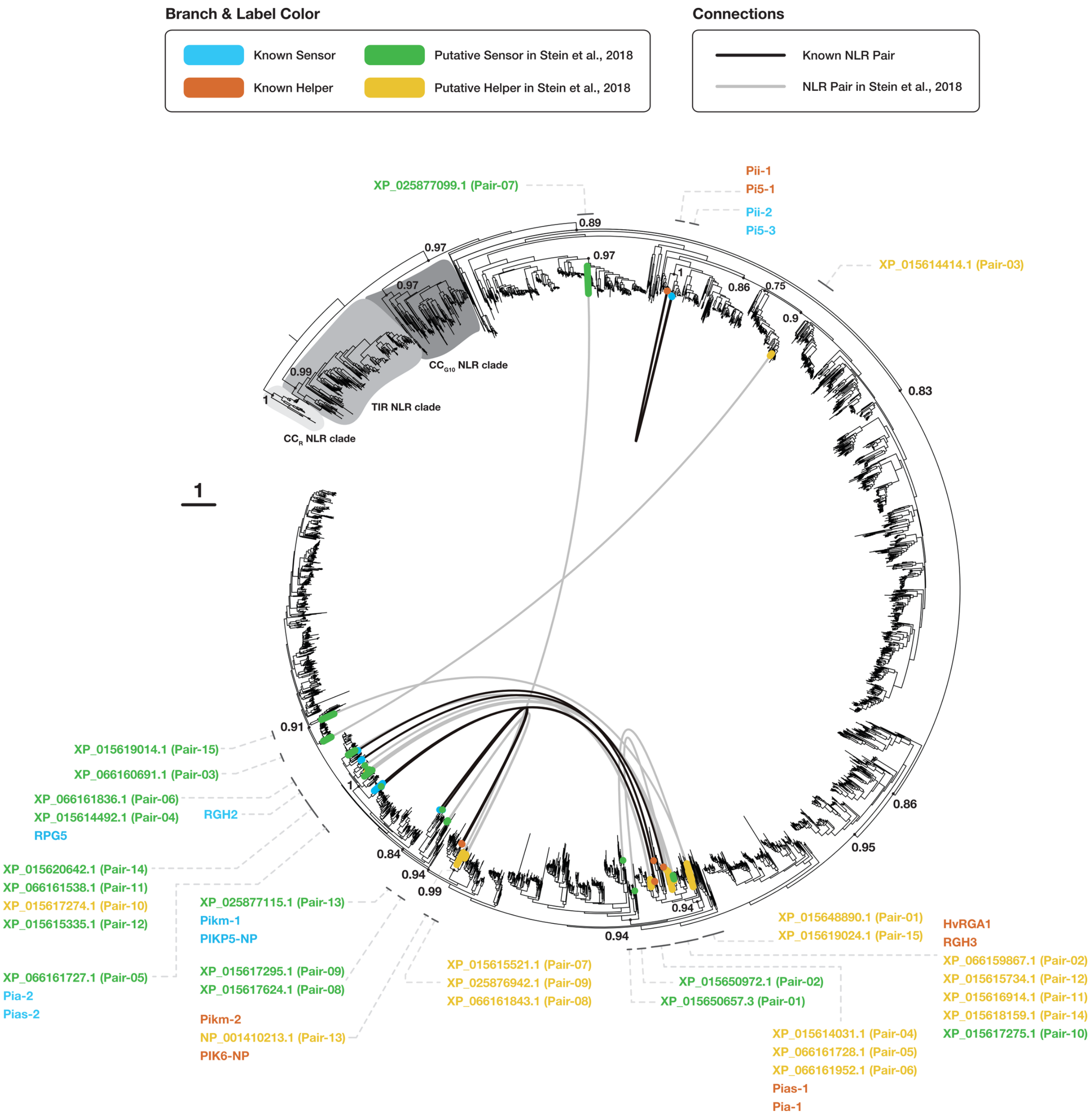

Fig. S1 Phylogenetic tree of monocot paired NLRs with RefPlantNLR and 4,936 NLR proteins from 13 RefSeq proteomes, including two dicot species and 11 monocot species obtained from Toghani et al., 2024. The tree was built using the NB-ARC domains of the eight known NLR pairs summarized in **Table S1**, RefPlantNLR, and 4,936 NLR proteins from the NLRtracker output of 13 RefSeq proteomes, including two dicot species (*Arabidopsis thaliana* and *Solanum*

lycopersicum) and 11 monocot species (*Zea mays*, *Triticum aestivum*, *Setaria viridis*, *Phragmites australis*, *Phoenix dactylifera*, *Oryza sativa*, *Musa acuminata*, *Lolium perenne*, *Hordeum vulgare* subsp. *vulgare*, *Brachypodium distachyon*, and *Asparagus officinalis*) (**Data S1, S2, S3, and S4** in https://github.com/amiralito/Paired_NLR_AF3) (Toghani & Kamoun, 2024). The paired NLRs of rice cultivar Nipponbare (GCF_034140825.1), described by Stein et al., 2018, are included among 13 RefSeq proteomes. The genetic linkage between NLR pairs is indicated by a connection between linked nodes. Known sensor and helper NLR nodes are colored blue and orange, respectively, while putative sensor and helper nodes are colored green and yellow. Numbers next to the tree nodes indicate bootstrap values. The tree is rooted at the CC_R-NLR clade. AlphaFold 3 was not performed for Pair-02, Pair-03, Pair-08, Pair-09, and Pair-10 because they do not carry a full N-terminal CC domain.

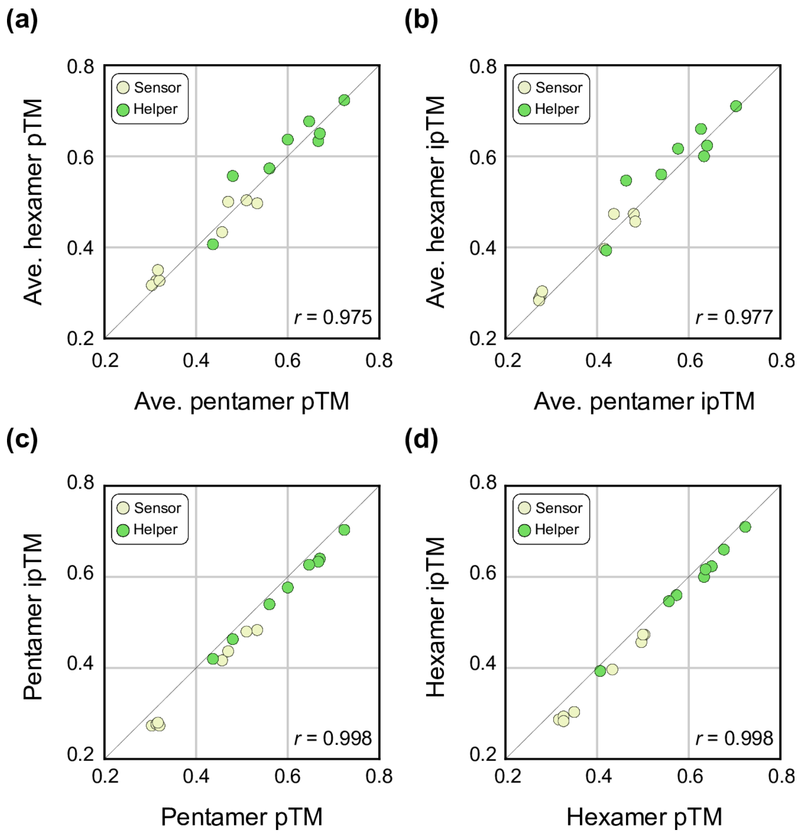

Fig. S2 Correlations between pentameric and hexameric AlphaFold 3 confidence scores and between pTM and ipTM scores for previously reported NLR pairs. a) Correlation between the average pentameric and hexameric pTM scores. b) Correlation between the average pentameric and hexameric ipTM scores. c) Correlation between the pentameric pTM and ipTM scores. d) Correlation between the hexameric pTM and ipTM scores. The amino acid sequences of the oligomerizing domains of the NLR proteins, from the N-terminus to the end of the NB-ARC domain, were used for the prediction. The pentameric and hexameric structures were modelled with 50 oleic acids using three different seed values. The resulting pTM or ipTM scores were averaged across three seed values for each NLR in Fig. S2a and S2b. The Pearson rank correlation coefficients (*r*) were calculated with the SciPy library in Python.

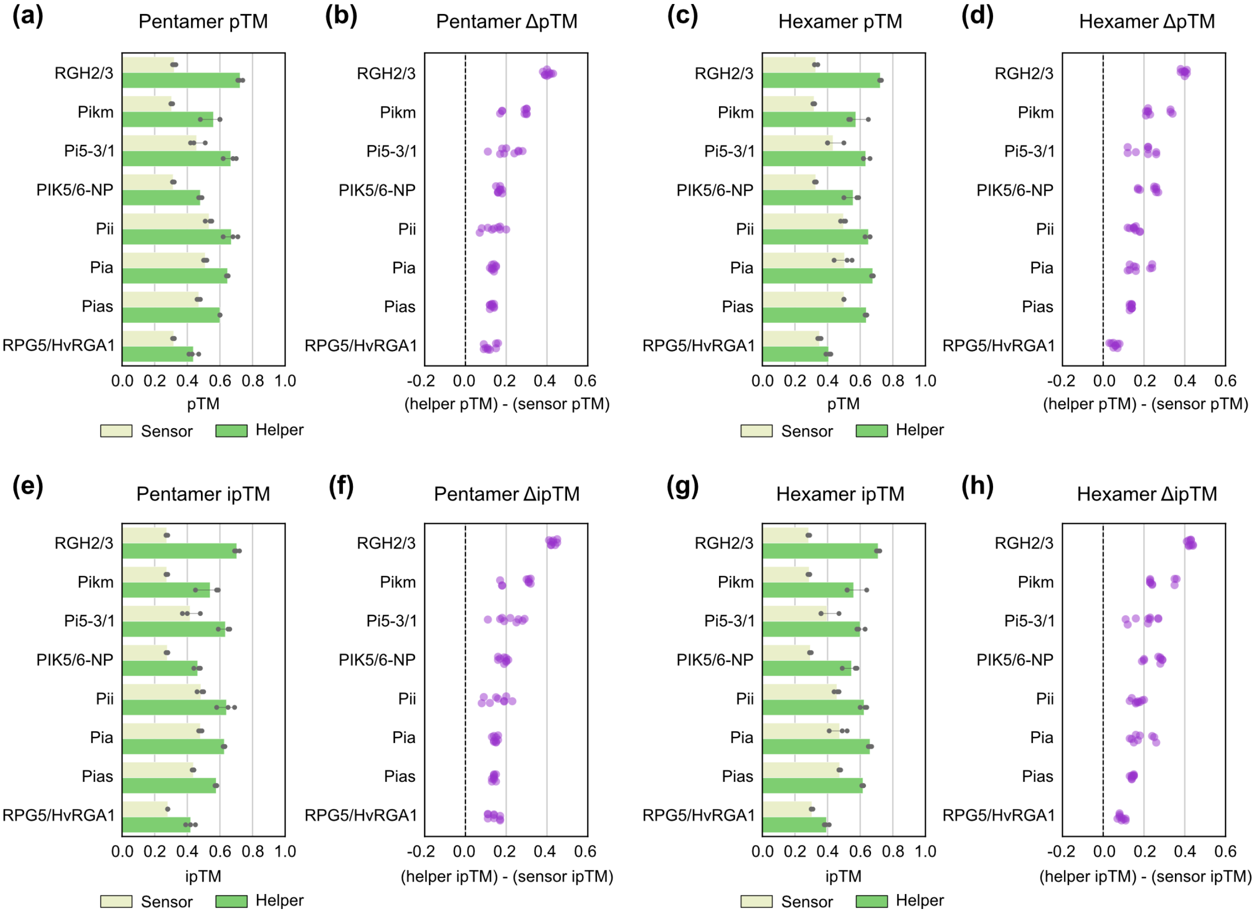

Fig. S3 Comparisons of sensor and helper AlphaFold 3 scores in pentameric and hexameric configurations for previously reported NLR pairs. a-d) pTM score comparisons for pentamers (a,b) and hexamers (c,d). e-h) ipTM score comparisons for pentamers (e,f) and hexamers (g,h). The a, c, e, and g panels show bar plots, and the b, d, f, and h panels display score differences (helper minus sensor). The amino acid sequences of the oligomerizing domains of the NLR proteins, from the N-terminus to the end of the NB-ARC domain, were used for the prediction. The pentameric and hexameric structures were modelled with 50 oleic acids using three different seed values. Subtractions were performed for all possible pairs of sensor and helper scores.

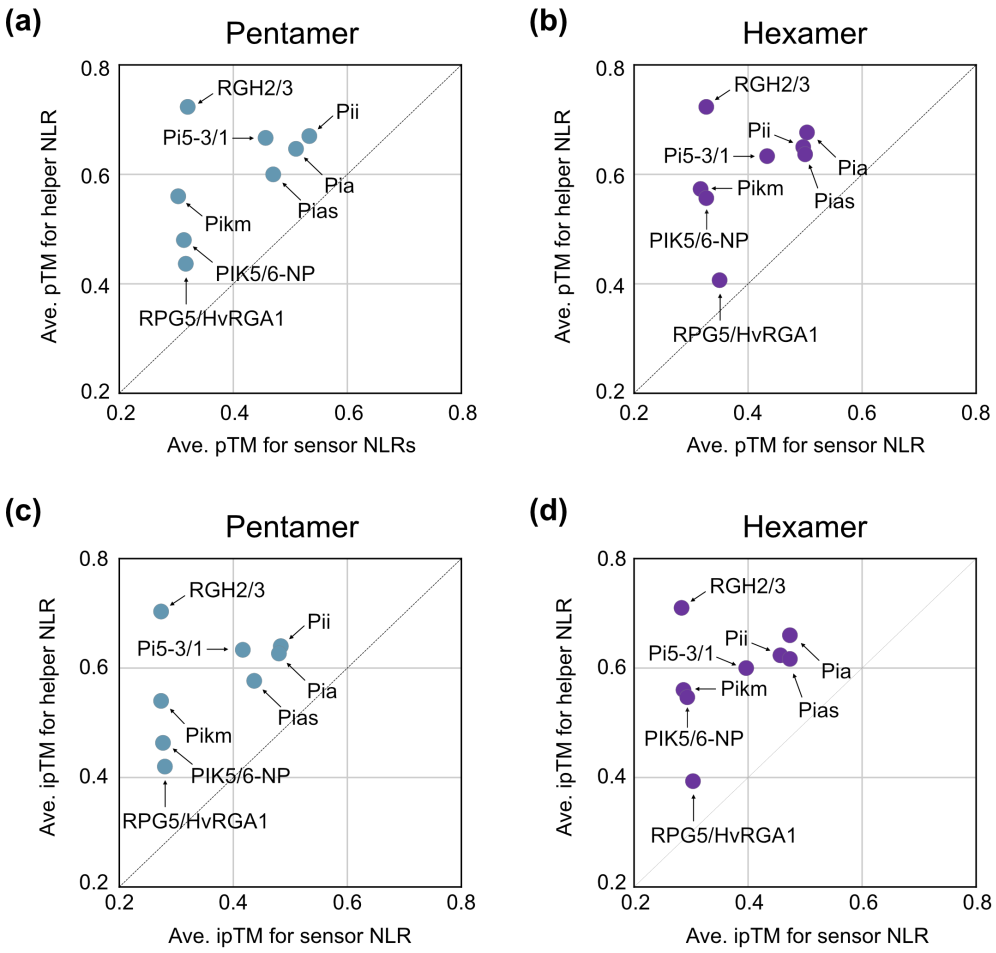

Fig. S4 Scatter plots comparing average sensor and helper scores in AlphaFold 3 predictions. a) Scatter plot comparing average sensor and helper pTM scores in pentamer predictions. b) Scatter plot comparing average sensor and helper pTM scores in hexamer predictions. c) Scatter plot comparing average sensor and helper ipTM scores in pentamer predictions. d) Scatter plot comparing average sensor and helper ipTM scores in hexamer predictions. The amino acid sequences of the oligomerizing domains of the NLR proteins, from the N-terminus to the end of the NB-ARC domain, were used for the prediction. The pentameric and hexameric structures were modelled with 50 oleic acids using three different seed values. The resulting pTM or ipTM scores were averaged across three seed values for each sensor and helper NLR.

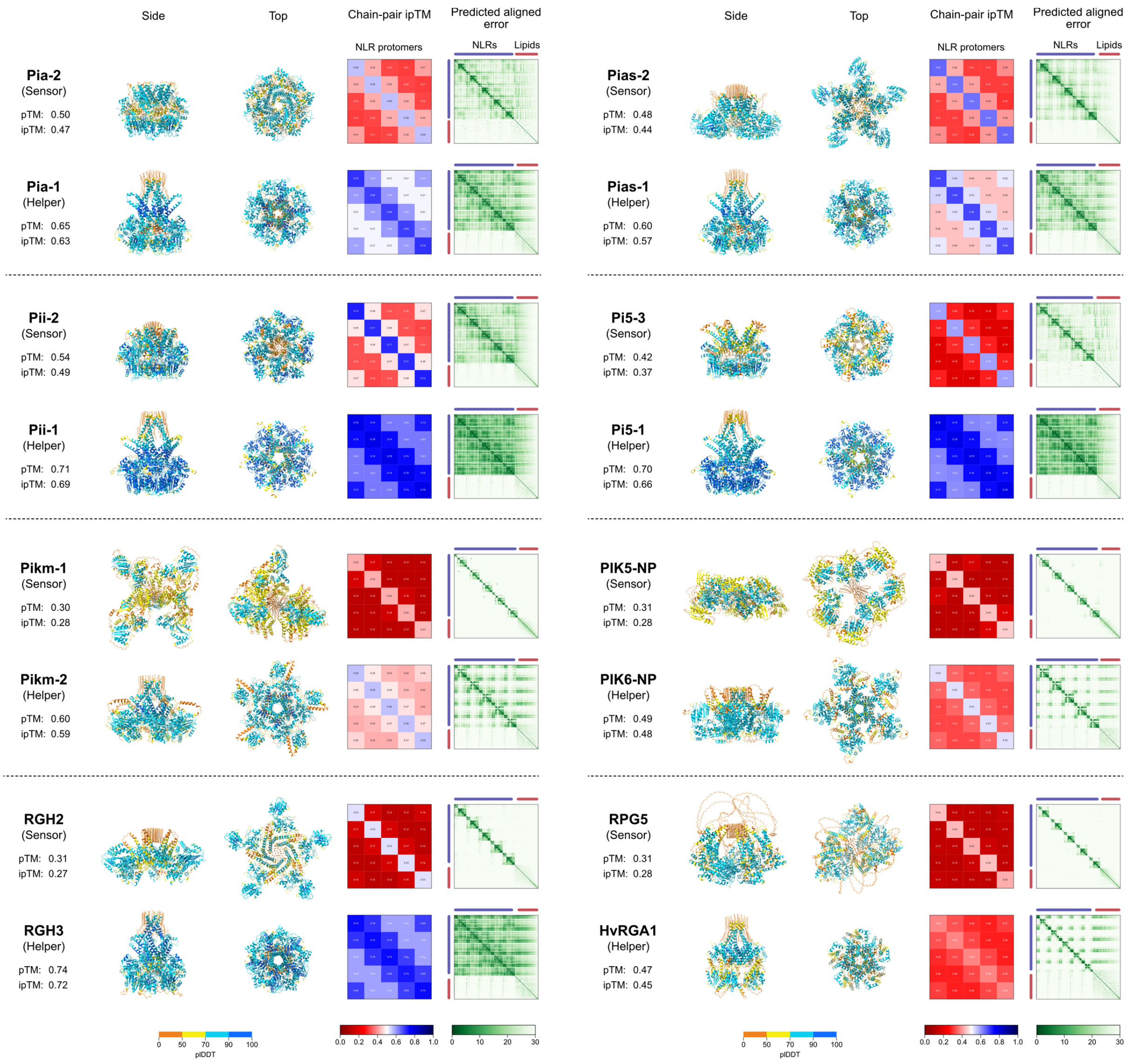

Fig. S5 Pentameric AlphaFold 3 predictions for previously reported NLR pairs. The amino acid sequences of the oligomerizing domains of the NLR proteins, from the N-terminus to the end of the NB-ARC domain, were used for the prediction. The pentameric structures were modelled with 50 oleic acids using seed value 1. The predicted structures were visualized with oleic acids (orange) using ChimeraX (Meng *et al.*, 2023).

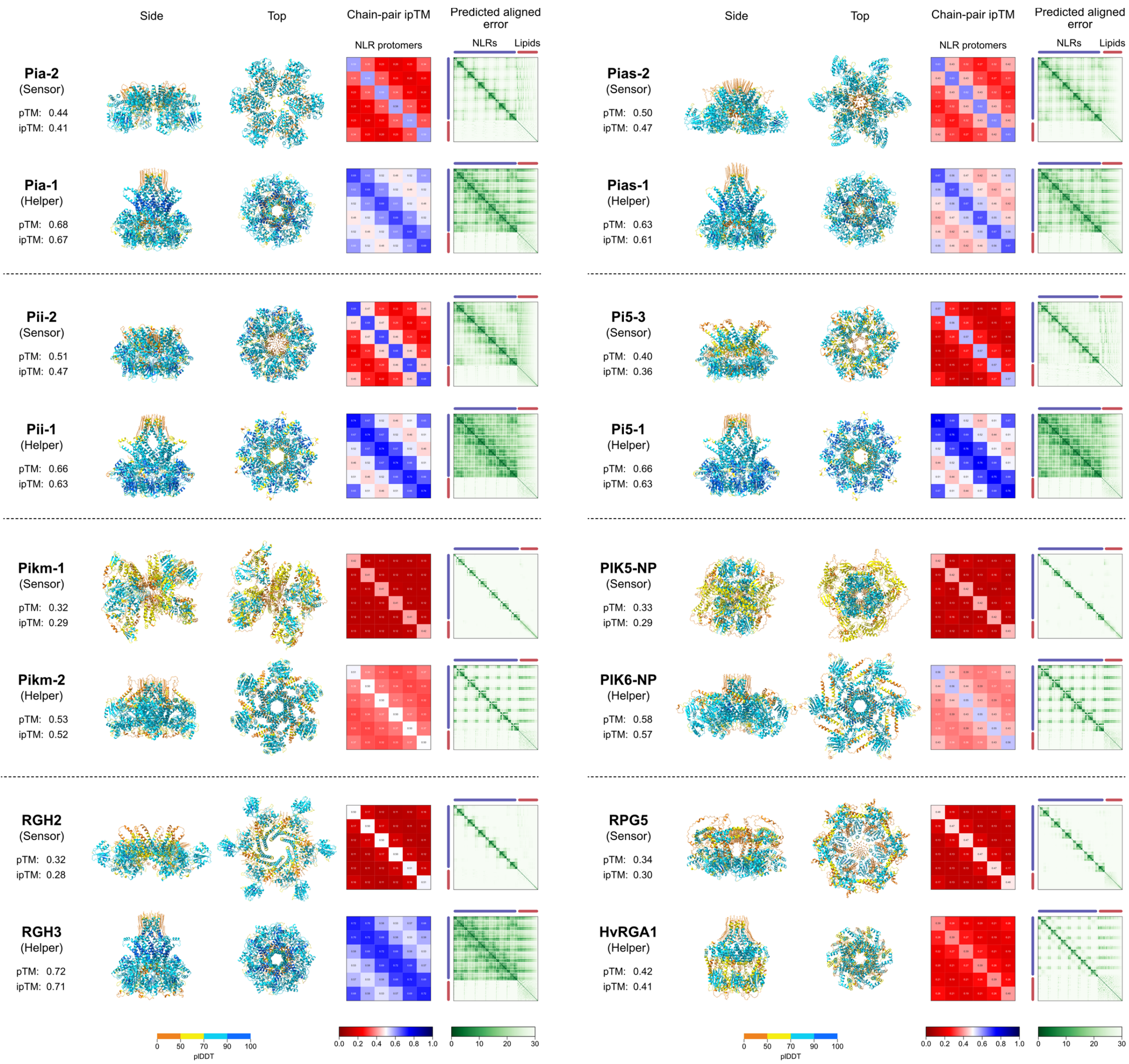

Fig. S6 Hexameric AlphaFold 3 predictions for previously reported NLR pairs. The amino acid sequences of the oligomerizing domains of the NLR proteins, from the N-terminus to the end of the NB-ARC domain, were used for the prediction. The hexameric structures were modelled with 50 oleic acids using seed value 1. The predicted structures were visualized with oleic acids (orange) using ChimeraX (Meng *et al.*, 2023).

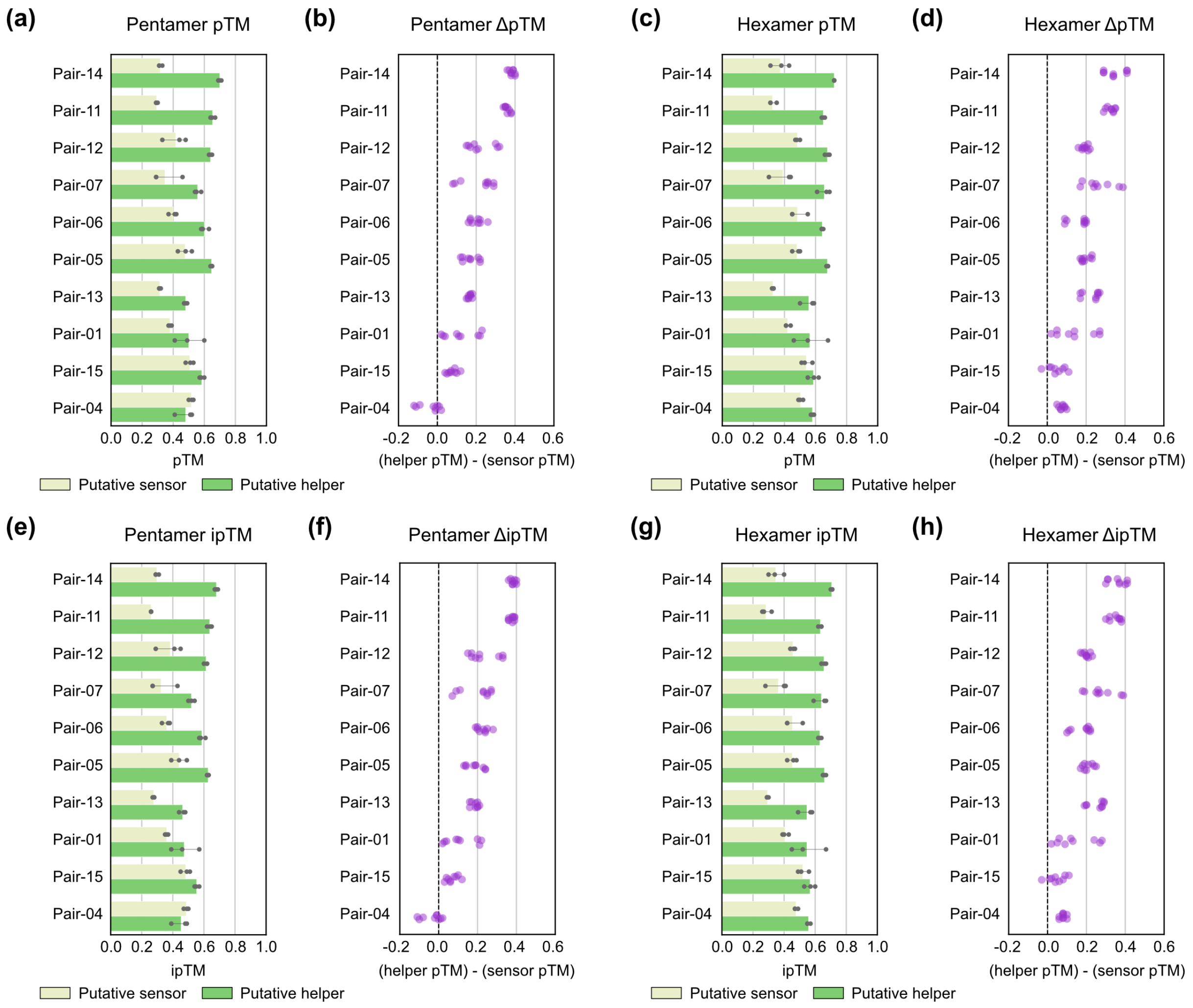

Fig. S7 Comparisons of putative sensor and helper AlphaFold 3 scores in pentameric and hexameric configurations for NLR pairs described by Stein et al., 2018. a-d) pTM score comparisons for pentamers (a,b) and hexamers (c,d). e-h) ipTM score comparisons for pentamers (e,f) and hexamers (g,h). The a, c, e, and g panels show bar plots, and the b, d, f, and h panels display score differences (putative helper minus putative sensor). The pentameric and hexameric structures were modelled with 50 oleic acids using three different seed values. Putative sensors and helpers were assigned based on the average pTM scores of pentameric and hexameric structures, with a putative sensor having the lower average score and a putative helper having the higher average score. Subtractions were performed for all possible pairs of putative sensor and helper scores.

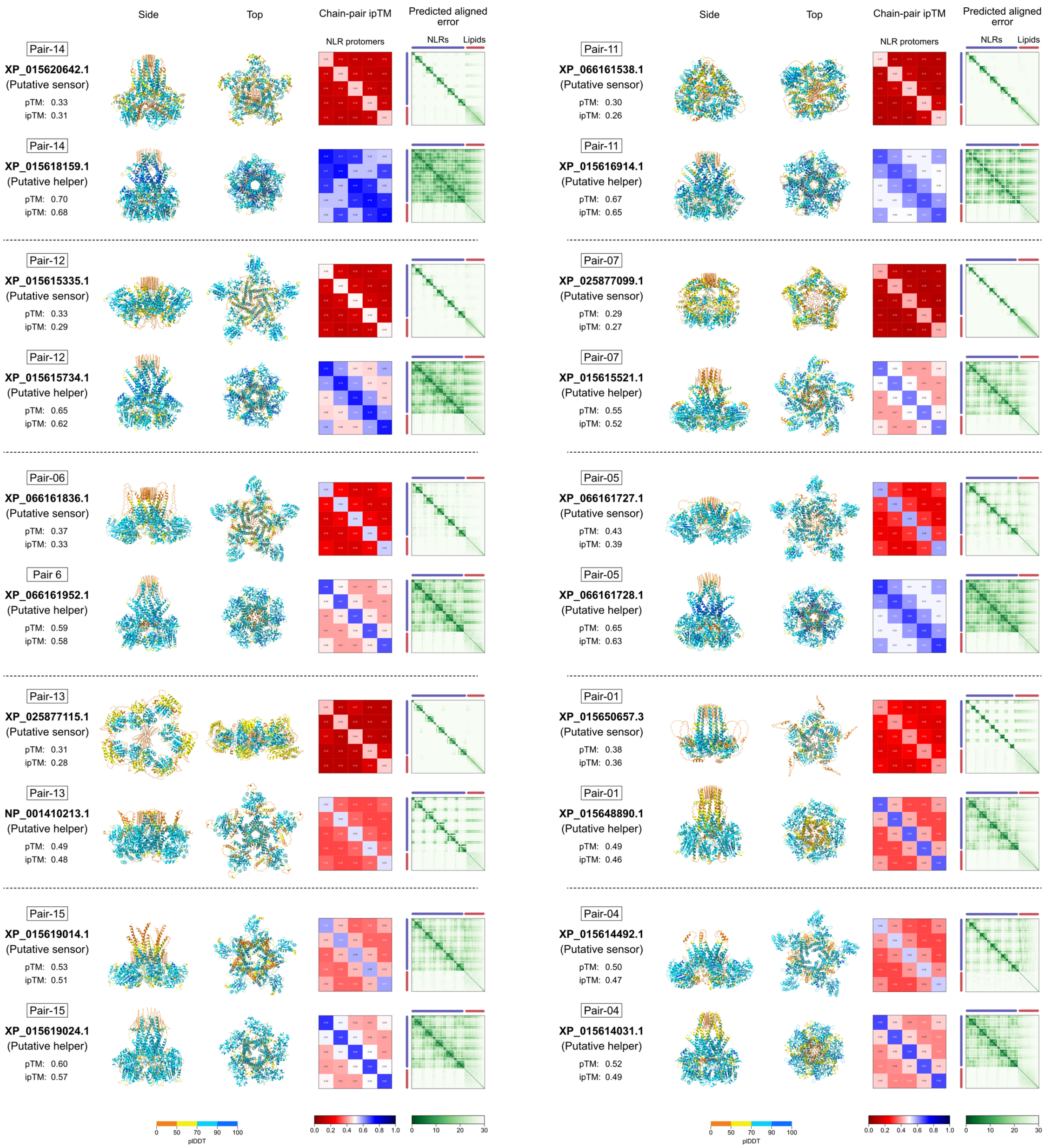

Fig. S8 Pentameric AlphaFold 3 predictions for NLR pairs described by Stein et al., 2018. The amino acid sequences of the oligomerizing domains of the NLR proteins, from the N-terminus to the end of the NB-ARC domain, were used for the prediction. The pentameric structures were modelled with 50 oleic acids using seed value 1. The predicted structures were visualized with oleic acids (orange) using ChimeraX (Meng *et al.*, 2023).

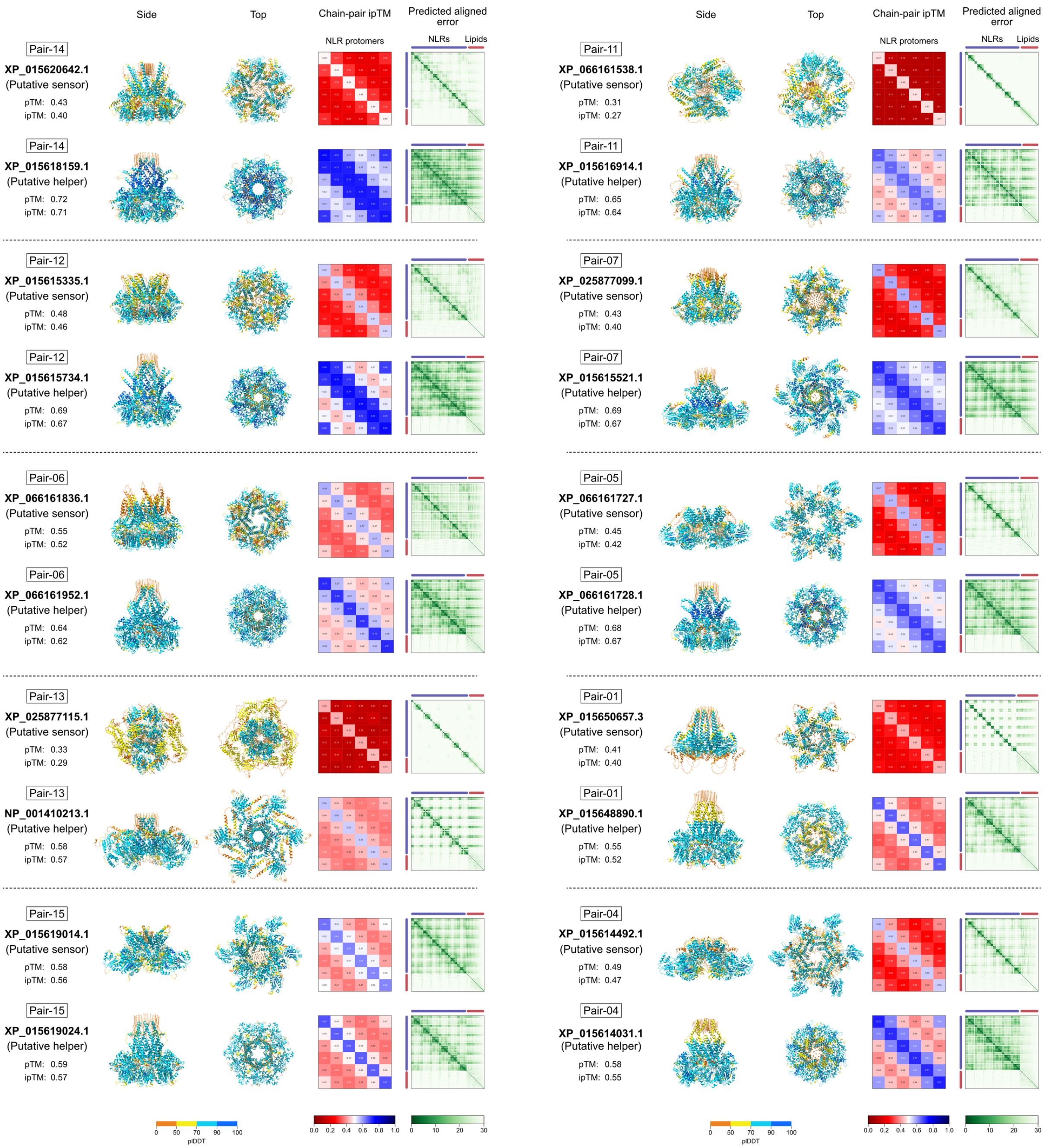

Fig. S9 Hexameric AlphaFold 3 predictions for NLR pairs described by Stein et al., 2018. The amino acid sequences of the oligomerizing domains of the NLR proteins, from the N-terminus to the end of the NB-ARC domain, were used for the prediction. The hexameric structures were modelled with 50 oleic acids using seed value 1. The predicted structures were visualized with oleic acids (orange) with ChimeraX (Meng *et al.*, 2023).

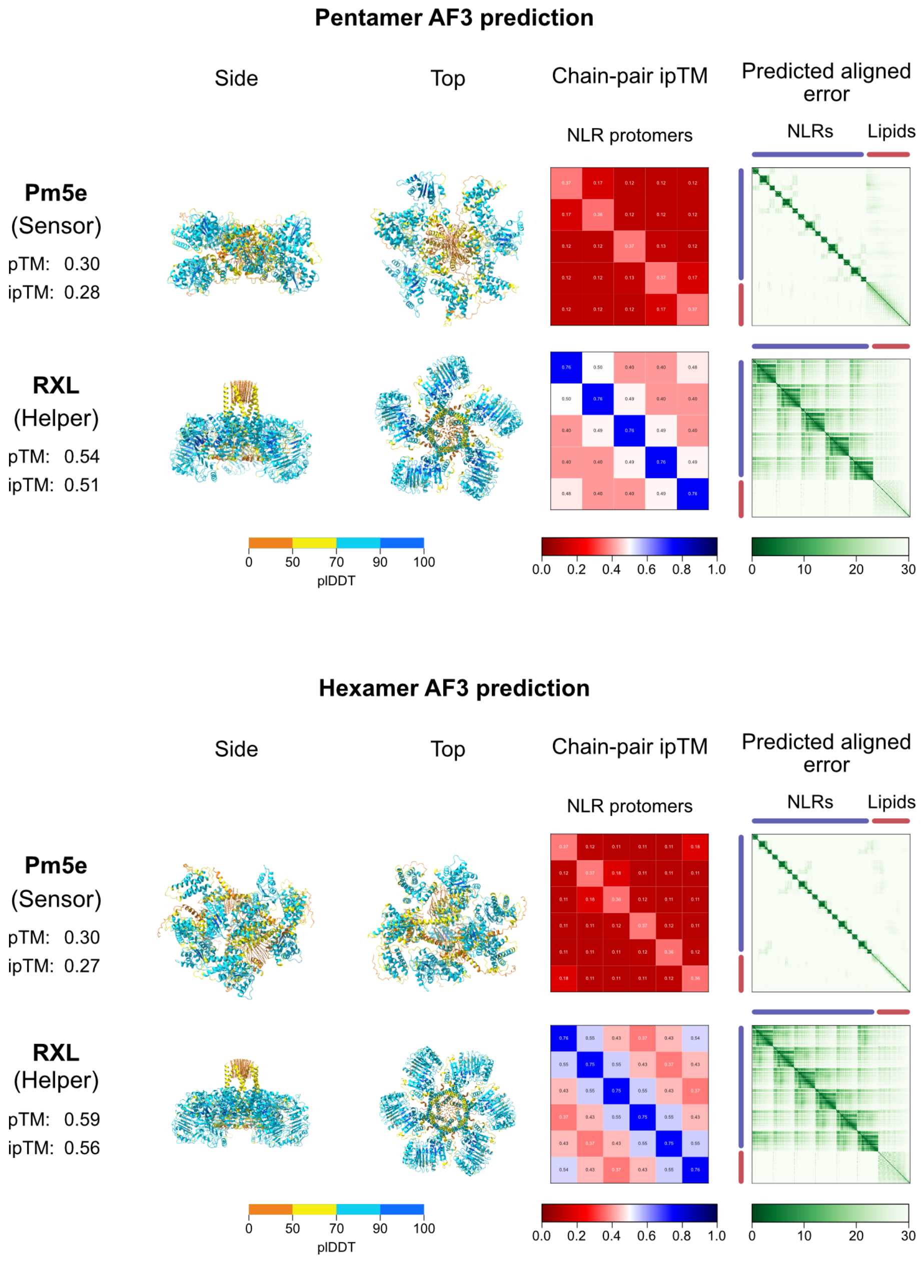

Fig. S10 Pentameric and hexameric AlphaFold 3 predictions of the wheat NLR pair Pm5e (sensor) and RXL (helper). The amino acid sequences of the oligomerizing domains of the NLR proteins, from the N-terminus to the end of the NB-ARC domain, was used for predicting the structure of Pm5e, while the full-length sequence of RXL was used due to its atypical domain architecture. The structures were modelled with 50 oleic acid molecules using seed value 1. The predicted structures, including the oleic acids (orange), were visualized using ChimeraX (Meng *et al.*, 2023).

See separate Excel file:

Table S1 List of previously reported NLR pairs. All sequences were derived from either RefPlantNLR (Kourelis et al., 2021) or NCBI. Sensor and helper NLRs are defined based on the presence or absence of an integrated domain, respectively. Domain architectures were annotated using NLRtracker (Kourelis et al., 2021). For domain architecture, "C", "N", "L", and "O" represent the CC, NB-ARC, LRR, and other integrated domains, respectively. Note that InterProScan did not annotate an integrated domain of PIK5-NP as previously reported (Kourelis et al., 2021). Therefore, we manually replaced the domain architecture of PIK5-NP from "CNL" to "CONL" as it contains the HMA domain between the NB-ARC and LRR (Białas et al., 2021).

Table S2 Summary of AlphaFold 3 predictions for previously reported NLR pairs.

Table S3 HMM scores of MADA motifs in previously reported NLR pairs. The presence or absence of the MADA or MADA-like motif was analyzed using the HMM model in Adachi et al., 2019. MADA, MADA-like, and no MADA motifs were classified based on HMM scores (> 10, < 10, and NA, respectively).

Table S4 List of rice NLR pairs described by Stein et al., 2018. The NLR pairs were identified from the rice cultivar Nipponbare genome annotation (Stein et al., 2018). Based on Supplementary Data 6 in Stein et al., 2018, the NLR pairs that i) are genetically linked in head-to-head orientations; ii) belong to distinct phylogenetic clades were extracted. Domain architectures were annotated using NLRtracker (Kourelis et al., 2021). Columns from Supplementary Data 6 in Stein et al., 2018 and those added in this study are highlighted in yellow and green, respectively.

Table S5 BLASTP results using NLRs from Stein et al., 2018 as queries against those in the NCBI RefSeq annotation as subjects. DIAMOND BLASTP (Buchfink et al., 2021) was used to identify corresponding sequences between the datasets. NLRs from Stein et al., 2018 and those in the

NCBI RefSeq annotation of the rice cultivar Nipponbare (GCF_034140825.1) served as the query and subject sets, respectively.

Table S6 Summary of NLRtracker outputs for rice NLR pairs from the NCBI RefSeq annotation of Nipponbare (GCF_034140825.1), corresponding to those described by Stein et al., 2018. Only NLR pairs with a full N-terminal CC domain in both NLRs were processed for AlphaFold 3 predictions.

Table S7 Summary of AlphaFold 3 predictions for rice NLR pairs from the NCBI RefSeq annotation of Nipponbare (GCF_034140825.1), corresponding to those described by Stein et al., 2018. Putative sensors and helpers were assigned based on the average pTM scores of pentameric and hexameric structures, with a putative sensor having the lower average score and a putative helper having the higher average score.

Table S8 Summary of the wheat NLR pair Pm5e (sensor) and RXL (helper) sequences. Domain architectures were annotated using NLRtracker (Kourelis et al., 2021). For domain architecture, "C", "N", and "L" represent the CC, NB-ARC, and LRR domains, respectively.

Table S9 Summary of AlphaFold 3 predictions for the wheat NLR pair Pm5e (sensor) and RXL (helper).

Adachi H, Contreras MP, Harant A, Wu C, Derevnina L, Sakai T, Duggan C, Moratto E, Bozkurt TO, Maqbool A, *et al.* 2019. An N-terminal motif in NLR immune receptors is functionally conserved across distantly related plant species (J-M Zhou, D Weigel, and J-M Zhou, Eds.). *eLife* **8**: e49956.

Białas A, Langner T, Harant A, Contreras MP, Stevenson CE, Lawson DM, Sklenar J, Kellner R, Moscou MJ, Terauchi R, *et al.* 2021. Two NLR immune receptors acquired high-affinity binding to a fungal effector through convergent evolution of their integrated domain (J Monaghan, J Kleine-Vehn, and P-M Delaux, Eds.). *eLife* **10**: e66961.

Buchfink B, Reuter K, Drost H-G. **2021**. Sensitive protein alignments at tree-of-life scale using DIAMOND. *Nature Methods* **18**: 366–368.

Kourelis J, Sakai T, Adachi H, Kamoun S. **2021**. RefPlantNLR is a comprehensive collection of experimentally validated plant disease resistance proteins from the NLR family. *PLOS Biology* **19**: e3001124.

Meng EC, Goddard TD, Pettersen EF, Couch GS, Pearson ZJ, Morris JH, Ferrin TE. **2023**. UCSF ChimeraX: Tools for structure building and analysis. *Protein Science* **32**: e4792.

Stein JC, Yu Y, Copetti D, Zwickl DJ, Zhang L, Zhang C, Chougule K, Gao D, Iwata A, Goicoechea JL, *et al.* 2018. Genomes of 13 domesticated and wild rice relatives highlight genetic conservation, turnover and innovation across the genus Oryza. *Nature Genetics* **50**: 285–296.

Toghani A, Kamoun S. **2024**. Functional annotation of 180 RefSeq reference plant proteomes reveals a dataset of 113,684 NLR proteins. doi:10.5281/zenodo.13627395.
